# Supplementary material for: Recurrent connectivity supports higher-level visual and semantic object representations in the brain
Source: Commun Biol. 2023 Nov 27;6:1207. doi: 10.1038/s42003-023-05565-9 (PMC10682037; doi:10.1038/s42003-023-05565-9)
Supplement: Supplementary file 1 — Supplementary material [file 42003_2023_5565_MOESM1_ESM.pdf]

Supplementary Figures:

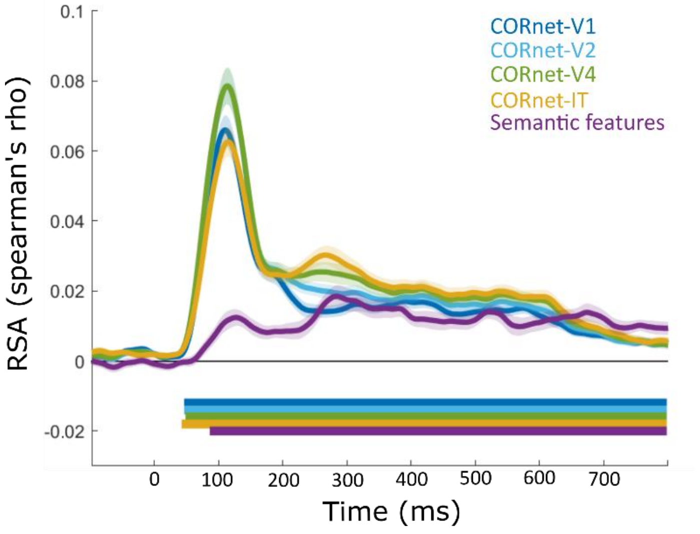

Supplementary Figure 1. RSA results for the MEG sensor array using non-partial correlation RSA analysis for each model RDM over time. Shaded areas show standard error of the mean. Solid bars show time periods of significant effects.

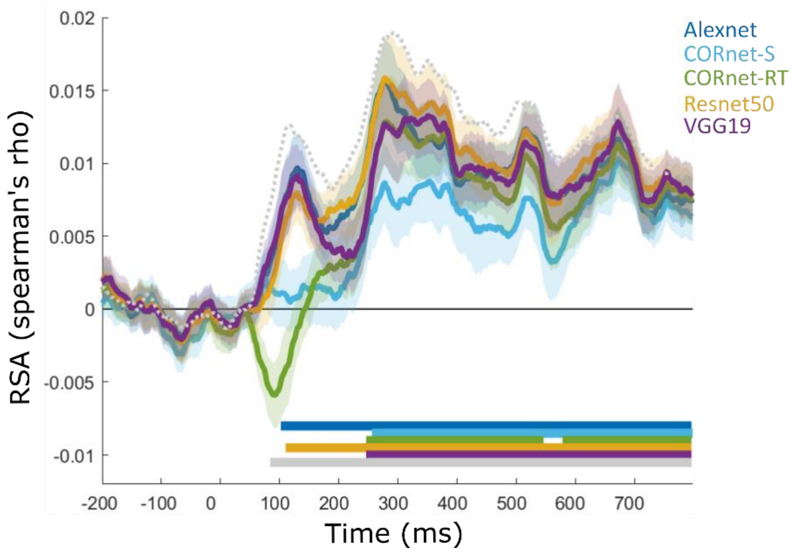

Supplementary Figure 2. RSA results for the semantic feature model after different ANNs have been partialled out. Shaded areas show standard error of the mean and dotted line shows the non-partial RSA results for the semantic feature model.

Supplementary tables:

Supplementary Table 1. fMRI searchlight results

Model RDM	*HO region*	*p(FWE)*	*k*	*T*	*voxel p*	*mm mm mm*
CORnet-V1	left inferior lateral occipital	0.0000	4497	11.981	0.0000	[-48;-70;-1]
	left temporal occipital fusiform			11.042	0.0000	[-39;-49;-20]
	right inferior lateral occipital			9.909	0.0000	[33;-85;-1]
CORnet-V2	left inferior lateral occipital	0.0000	6118	12.553	0.0000	[-45;-67;-5]
	right inferior lateral occipital			11.940	0.0000	[33;-85;-1]
	left temporal occipital fusiform			10.491	0.0000	[-39;-49;-20]
	right precentral	0.0018	177	5.834	0.0000	[36;-7;44]
	right postcentral			5.208	0.0001	[33;-34;51]
	right superior parietal			5.200	0.0001	[36;-55;51]
CORnet-V4	left inferior lateral occipital	0.0000	7352	12.148	0.0000	[-48;-73;-1]
	right inferior lateral occipital			11.796	0.0000	[36;-82;3]
	right temporal occipital fusiform			9.697	0.0000	[33;-49;-9]
	left postcentral	0.0137	121	6.161	0.0000	[-54;-25;44]
	left supramarginal			5.188	0.0001	[-51;-34;33]
CORnet-IT	left inferior lateral occipital	0.0000	5711	10.529	0.0000	[-45;-70;-1]
	right inferior lateral occipital			8.870	0.0000	[45;-76;-13]
	right inferior lateral occipital			8.739	0.0000	[33;-88;-1]
	left postcentral	0.0023	181	6.971	0.0000	[-54;-25;44]
	left postcentral			5.316	0.0000	[-39;-31;48]
Semantic features	right temporal occipital fusiform	0.0000	4151	11.149	0.0000	[30;-49;-13]
	right inferior lateral occipital			8.674	0.0000	[33;-88;-1]
	right temporal occipital fusiform			8.397	0.0000	[24;-40;-20]
	left supramarginal	0.0011	206	7.146	0.0000	[-51;-34;36]
	left postcentral			4.173	0.0004	[-66;-19;29]
Semantic features (partial)	right temporal occipital fusiform	0.0000	1268	12.072	0.0000	[30;-49;-13]
	right temporal occipital fusiform			8.203	0.0000	[24;-40;-20]
	right temporal posterior fusiform			7.363	0.0000	[36;-34;-16]
	left temporal occipital fusiform	0.0000	564	7.570	0.0000	[-30;-55;-9]
	left lingual			6.492	0.0000	[-21;-58;-5]
	left inferior lateral occipital			5.976	0.0000	[-45;-67;-1]
	right precuneus	0.0012	198	7.305	0.0000	[6;-55;36]
	left precuneous			4.747	0.0001	[-9;-49;44]
	right precuneus			4.329	0.0003	[9;-64;25]
	left supramarginal	0.0199	108	6.121	0.0000	[-51;-34;36]

Coordinates in MNI space, and region labels from the Harvard-Oxford Atlas

Supplementary Table 2. RSA sensor effects for the partial correlation analysis

Model RDM	*Model Peak (95% CIs)*	*Time window*	*cluster mass*	*cluster p*
CORnet-V1	100 (94-102)	'85-114 ms'	54.53	0.0463
		'355-414 ms'	89.13	0.0162
CORnet-V2	120 (120-128)	'109-154 ms'	93.93	0.0108
CORnet-V4	96 (88-102)	'67-140 ms'	133.42	0.0032
CORnet-IT	258 (256-298)	'131-602 ms'	891.53	<0.0001
		'607-646 ms'	53.83	0.0462
Semantic features	352 (274-382)	'259-400 ms'	217.76	0.0011
		'491-538 ms'	79.22	0.0228
		'595-802 ms'	396.77	0.0002

Supplementary Table 3. RSA sensor effects for the semantic feature model controlling for different ANNs

Control model RDMs	*Time window*	*cluster mass*	*cluster p*
Alexnet	'103-802 ms'	1540.58	0.0001
CORnet-S	'259-400 ms'	217.76	0.0011
	'491-538 ms'	79.22	0.0228
	'595-802 ms'	396.77	0.0002
CORnet-RT	'249-548 ms'	627.15	0.0002
	'581-802 ms'	458.87	0.0003
Resnet50	'111-802 ms'	1549.88	<0.0001
VGG19	'103-174 ms'	122.87	0.0103
	'249-802 ms'	1205.94	<0.0001
No control	'73-802 ms'	2092.03	<0.0001

Supplementary Table 4. RCA results showing feedforward (FF) and feedback (FB) effects

Model RDM	*Direction*	*Model Peak (95% CIs)*	*Time window*	*cluster mass*	*cluster p*
CORnet-V1	FF	110 (106-112)	'89-140 ms'	114.86	0.0032
	FF		'177-218 ms'	71.98	0.0156
	FF		'381-442 ms'	97.84	0.0065
	FF		'577-612 ms'	63.18	0.0209
	FF		'627-670 ms'	70.35	0.0165
CORnet-V2	FF	138 (132-138)	'117-166 ms'	127.78	0.0003
CORnet-V4	FF	112 (106-116)	'75-178 ms'	287.30	0.0002
	FF		'237-388 ms'	257.31	0.0003
	FF		'421-472 ms'	76.01	0.0141
	FF		'537-582 ms'	67.91	0.0195
	FF		'605-648 ms'	71.10	0.0180
CORnet-IT	FF	260 (258-274)	'75-716 ms'	1660.74	<0.0001
Semantic features	FF	298 (298-318)	'273-802 ms'	937.33	<0.0001
CORnet-IT	FB	334 (262-374)	'243-408 ms'	306.26	0.0003
	FB		'501-536 ms'	53.75	0.0273
Semantic features	FB	398 (384-422)	'383-416 ms'	52.71	0.0276
	FB		'523-552 ms'	52.17	0.0285
	FB		'601-802 ms'	358.31	<0.0001

Supplementary Table 5. RCA results for the semantic model RDM

	Model peak (95% CI)	Time window	cluster mass	cluster p
pVTC to right ATL	324 (300-326)	'147-802 ms'	1338.48	< 0.001
pVTC to left PFC/ATL	302 (290-310)	'147-416 ms'	497.84	< 0.001
pVTC to left PFC/ATL		'555-632 ms'	109.14	0.009
pVTC to left PFC/ATL		'643-802 ms'	235.69	0.001
Right ATL to pVTC	402 (386-448)	'385-462 ms'	116.44	0.0058
Left PFC/ATL to pVTC	290 (284-470)	'441-496 ms'	97.11	0.0071
Left PFC/ATL to pVTC		'601-656 ms'	91.09	0.0090
Left PFC/ATL to right ATL	462 (390-472)	'439-482 ms'	83.85	0.0078

Supplementary Table 6. RCA results for visual and semantic model RDMs

Model RDM	Direction	Peak time (95% CI)	Time window	cluster mass	cluster p
CORnet-V1	pVTC to right ATL	116 (114-116)	'93-154 ms'	130.79	0.006
	pVTC to right ATL		'335-438 ms'	160.85	0.003
CORnet-V2	pVTC to right ATL	134 (134-134)	'103-216 ms'	223.45	0.002
CORnet-V4	pVTC to right ATL	114 (102-148)	'69-400 ms'	772.08	0.000
CORnet-IT	pVTC to right ATL	278 (122-288)	'81-756 ms'	1690.44	0.000
Semantic features	pVTC to right ATL	324 (300-326)	'147-802 ms'	1338.48	0.000
CORnet-V1	pVTC to left PFC/ATL	116 (110-118)	'93-150 ms'	111.39	0.004
CORnet-V2	pVTC to left PFC/ATL	136 (126-136)	'75-200 ms'	192.36	0.001
CORnet-V4	pVTC to left PFC/ATL	136 (104-136)	'75-268 ms'	364.23	0.000
	pVTC to left PFC/ATL		'289-478 ms'	338.55	0.000
CORnet-IT	pVTC to left PFC/ATL	118 (116-136)	'79-754 ms'	1248.74	0.000
Semantic features	pVTC to left PFC/ATL	302 (290-310)	'147-416 ms'	497.84	0.000
	pVTC to left PFC/ATL		'555-632 ms'	109.14	0.009
	pVTC to left PFC/ATL		'643-802 ms'	235.69	0.001
CORnet-IT	Right ATL to pVTC	282 (246-290)	'169-324 ms'	312.79	0.0006
	Right ATL to pVTC		'347-418 ms'	103.05	0.0103
Semantic features	Right ATL to pVTC	402 (386-448)	'385-462 ms'	116.44	0.0058
	Left PFC/ATL to pVTC		'441-496 ms'	97.11	0.0071
	Left PFC/ATL to pVTC		'601-656 ms'	91.09	0.0090
	Left PFC/ATL to right ATL		'439-482 ms'	83.85	0.0078
