# Supplementary material for: Recurrent connectivity supports higher-level visual and semantic object representations in the brain
Source: Commun Biol. 2023 Nov 27;6:1207. doi: 10.1038/s42003-023-05565-9 (PMC10682037; doi:10.1038/s42003-023-05565-9)
Supplement: Supplementary file 2 — Reporting summary [file 42003_2023_5565_MOESM2_ESM.pdf]

nature portfolio

Corresponding author(s): Alex Clarke

Last updated by author(s): Sep 12, 2023

Reporting Summary

Nature Portfolio wishes to improve the reproducibility of the work that we publish. This form provides structure for consistency and transparency in reporting. For further information on Nature Portfolio policies, see our Editorial Policies and the Editorial Policy Checklist.

Statistics

For all statistical analyses, confirm that the following items are present in the figure legend, table legend, main text, or Methods section.

n/a	Confirmed	
☐	☒	The exact sample size (*n*) for each experimental group/condition, given as a discrete number and unit of measurement
☐	☒	A statement on whether measurements were taken from distinct samples or whether the same sample was measured repeatedly
☐	☒	The statistical test(s) used AND whether they are one- or two-sided<br>*Only common tests should be described solely by name; describe more complex techniques in the Methods section.*
☐	☒	A description of all covariates tested
☐	☒	A description of any assumptions or corrections, such as tests of normality and adjustment for multiple comparisons
☐	☒	A full description of the statistical parameters including central tendency (e.g. means) or other basic estimates (e.g. regression coefficient) AND variation (e.g. standard deviation) or associated estimates of uncertainty (e.g. confidence intervals)
☐	☒	For null hypothesis testing, the test statistic (e.g. *F*, *t*, *r*) with confidence intervals, effect sizes, degrees of freedom and *P* value noted<br>*Give P values as exact values whenever suitable.*
☒	☐	For Bayesian analysis, information on the choice of priors and Markov chain Monte Carlo settings
☒	☐	For hierarchical and complex designs, identification of the appropriate level for tests and full reporting of outcomes
☐	☒	Estimates of effect sizes (e.g. Cohen's *d*, Pearson's *r*), indicating how they were calculated

Our web collection on statistics for biologists contains articles on many of the points above.

Software and code

Policy information about availability of computer code

Data collection	Stimuli were presented using Eprime version 2
Data analysis	All data analysis was conducted in Matlab using different toolboxes:<br>For the fMRI analysis, Matlab was used along with the RSA toolbox (Nili et al., 2014) hosted here: https://www.mrc-cbu.cam.ac.uk/methods-and-resources/toolboxes/license/. For the MEG analysis, Matlab was also used. SPM12 was used for the preprocessing of the data. RSA analyses were conducted using a custom toolbox, and source-level searchlight analysis using the custom toolbox, both found here (https://github.com/AlexDClarke).

For manuscripts utilizing custom algorithms or software that are central to the research but not yet described in published literature, software must be made available to editors and reviewers. We strongly encourage code deposition in a community repository (e.g. GitHub). See the Nature Portfolio guidelines for submitting code & software for further information.

Data

Policy information about availability of data

All manuscripts must include a data availability statement. This statement should provide the following information, where applicable:

- Accession codes, unique identifiers, or web links for publicly available datasets
- A description of any restrictions on data availability
- For clinical datasets or third party data, please ensure that the statement adheres to our policy

The data used in this research was obtained from different experiments with different availabilities. The MEG data collected as part of the Cambridge Centre for Ageing and Neuroscience was part of stage III and is available upon requested (see https://camcan-archive.mrc-cbu.cam.ac.uk/dataaccess/). The remaining MEG data is found here https://osf.io/2uqf4/, and fMRI data here https://osf.io/e2s59/.

Human research participants

Policy information about studies involving human research participants and Sex and Gender in Research.

Reporting on sex and gender	Neither biological sex or gender was considered in the study design, and was not a factor that contributed to the data analysis. The results we report are applicable to all genders and sex.
Population characteristics	The age range of the participants was 18-37, although this was not added as a covariate in the analysis.
Recruitment	Participants were recruited through opportunity sampling of the local population, via adverts placed in public spaces (e.g. libraries). Participants were required to meet the eligibility criteria for neuroimaging scanning, and were in the age range 18-37. The sample is likely biased towards the Cambridge student population, although we do not think this would have any impact on the results we report.
Ethics oversight	Informed consent was obtained from all participants and ethical approval for the studies were obtained from the Cambridgeshire Research Ethics Committee. All experiments were performed in accordance with relevant guidelines and regulations.

Note that full information on the approval of the study protocol must also be provided in the manuscript.

Field-specific reporting

Please select the one below that is the best fit for your research. If you are not sure, read the appropriate sections before making your selection.

☐ Life sciences ☒ Behavioural & social sciences ☐ Ecological, evolutionary & environmental sciences

For a reference copy of the document with all sections, see nature.com/documents/nr-reporting-summary-flat.pdf

Behavioural & social sciences study design

All studies must disclose on these points even when the disclosure is negative.

Study description	The study is multi-part and quantitative, involving an fMRI study and an MEG study. In both studies, participants performed a picture naming task with common objects and animals while either fMRI or MEG was obtained. Separate groups of participants were engaged in the two studies.
Research sample	The age range of participants was 18-29 in the fMRI study and 18-37 in the MEG study. This came from existing datasets (1) fMRI from Clarke & Tyler (2014) published in the Journal of Neuroscience. The participants were from the local Cambridge population. (2) 15 participants took part in MEG that was twice previously published as Clarke et al., (2015) Cerebral Cortex, and Clarke et al., (2018), Journal of Cognitive Neuroscience. (3) An additional 21 participants were selected form the Cam-CAN dataset who had performed the same task, with the sample coming from the local Cambridge population, and previously published as Bruffaerts et al., (2019), Scientific Reports.
Sampling strategy	For the fMRI data, the sample size was chosen to match other similar studies collected at the time (in 2012). The dataset has been previsouly published, using similar techniques to here suggesting it has enough power. For the MEG study, we had the opportunity to combine studies that used the same items and methodology, but were collected through different groups. This gave a sample size of 36 for the MEG study, which is larger than most neuroimaging studies to date.
Data collection	fMRI: Participants were scanned at the MRC Cognition and Brain Sciences Unit, Cambridge, in a Siemens 3-T Tim Trio MRI scanner (Siemens Medical Solutions, Camberley, UK).<br><br>MEG: All MEG data were collected at the MRC Cognition and Brain Sciences Unit, Cambridge, UK, using a whole-head 306 channel (102 magnetometers, 204 planar gradiometers) Vector-view system (Elekta Neuromag, Helsinki, Finland). Blinks and eye movements

	were recorded using electro-oculogram (EOG) electrodes and the head position was recorded using five Head-Position Indicator (HPI) coils. Participants' head shapes, and the positions of EOG electrodes, HPI coils and fiducial points (nasion, left and right periauricular) were digitally recorded with a 3D digitiser (Fastrak Polhemus, Inc., Colchester, VA, USA).<br><br>In all sessions, the participant, research and imaging facilities operator was present. The study does not involve different groups, and each item shown to the participant is a separate condition (fMRI 131 conditions, MEG 302 conditions).
Timing	All the data is reused from previous studies meaning exact dates are hard to know. The fMRI data was collected in 2012, the MEG dataset from Clarke et al., (2015) was collected in 2013, and the MEG data from Bruffaerts t al., (2019)/phase III of Cam-CAN project which launched in 2010.
Data exclusions	No exclusions
Non-participation	No participant declined participation
Randomization	Participants were no allocated into groups

Reporting for specific materials, systems and methods

We require information from authors about some types of materials, experimental systems and methods used in many studies. Here, indicate whether each material, system or method listed is relevant to your study. If you are not sure if a list item applies to your research, read the appropriate section before selecting a response.

Materials & experimental systems

n/a	Involved in the study
☒	☐ Antibodies
☒	☐ Eukaryotic cell lines
☒	☐ Palaeontology and archaeology
☒	☐ Animals and other organisms
☒	☐ Clinical data
☒	☐ Dual use research of concern

Methods

n/a	Involved in the study
☒	☐ ChIP-seq
☒	☐ Flow cytometry
☐	☒ MRI-based neuroimaging

Magnetic resonance imaging

Experimental design

Design type	Participants performed a picture naming task during in an event related design
Design specifications	The fMRI study used 145 object images, that were each repeated six times. Each of the 145 objects was presented once during each scanning block, with the order of items in each block randomised. A different randomisation was used for each participant. Each trial/object begain with a 500 ms fixation cross, followed by presentation of the object for 500 ms, followed by a blank screen lasting for a random interval between 3 and 11 seconds.
Behavioral performance measures	The naming response was recorded by the experimenter during the session, and a sound recording was used to verify the accuracy of responses.

Acquisition

Imaging type(s)	Functional
Field strength	3T
Sequence & imaging parameters	Gradient-echo echoplanar imaging sequences collecting 32 slices in descending order of 3 mm thickness and between slice gap of 0.75 mm, and a resolution of 3 x 3 mm. The field-of-view was 192 x 192 mm, matrix size 64 x 64 with a TR of 2 s, TE of 30 ms, and a flip angle of 78°.
Area of acquisition	Whole brain

Diffusion MRI ☐ Used ☒ Not used

Preprocessing

Preprocessing software	Preprocessing consisted of slice-time correction and the spatial realignment of the functional images only using SPM8 (Wellcome Institute of Cognitive Neurology, London, UK). The resulting unsmoothed, un-normalized data for each participant were analyzed using the general linear model to create a single image for each object based on all six repetitions that were used to create single object t-statistic maps. In addition to the 145 object predictors, predictors were included to capture slow trends using 18 regressors for each session based on the basis functions of a discrete cosine transform (minimum

	frequency 1/128 Hz), six head motion regressors for each session, and a global mean predictor for each scanning session. Before fMRI scanning, participants received instructions and practice of how to name objects during the scanning session to reduce any potential motion artifacts. Further we examined the realignment parameters to ensure head motion was not in excess of 3 mm in any direction during a session which was the case for 13 of the participants. The remaining three participants had motion not in excess of 4 mm in any direction.
Normalization	The RSA analysis was performed on un-normalised data to preserve the voxels patterns for the analysis. The RSA correlation maps for each participant were normalised to the MNI template space and spatially smoothed using a 6 mm FWHM Gaussian kernel.
Normalization template	MNI template in SPM.
Noise and artifact removal	Predictors were included in the GLMs to capture slow trends using 18 regressors for each session based on the basis functions of a discrete cosine transform (minimum frequency 1/128 Hz), six head motion regressors for each scanning session, and a global mean predictor for each scanning session.
Volume censoring	RSA analysis was restricted to grey matter voxels. To define a voxel as grey matter, we set a threshold of >0.2 applied to the segmented structural MRI image.

Statistical modeling & inference

Model type and settings	RSA: At each voxel, object activation values from gray matter voxels within a spherical searchlight (radius 7 mm, maximum dimensions 5 x 5 x 3 voxels) were extracted to calculate distances between all objects (using 1 - Pearson's correlation) creating an object dissimilarity matrix based on that searchlight. This fMRI RDM was then compared with each theoretical model RDM (using Spearman's rank correlation) and the resulting similarity values were Fisher transformed and mapped back to the voxel at the center of the searchlight. For group random-effects analyses, the Spearman's correlation maps for each participant were Fisher-transformed, normalized to standard MNI space, and spatially smoothed with a 6mm FWHM Gaussian kernel. Maps were entered into a random effects analysis (RFX) in SPM12 where each voxel was tested using a one-sampled t-test against zero. Voxelwise multiple comparisons correction was applied through a voxelwise threshold of $p < 0.001$ and FWE-cluster $p < 0.05$.
Effect(s) tested	RSA correlations between brain-based RDMs and model RDMs created from (1) CORnet-S (a four-layer recurrent DNN; Kubilius et al., 2019) and (2) a property-norming study (Devereux et al., 2014). Each model was tested using one-sampled t-tests against zero at each voxel, with SPM cluster correction used to control for multiple comparisons.

Specify type of analysis: ☒ Whole brain ☐ ROI-based ☐ Both

Statistic type for inference (See Eklund et al. 2016)	Voxelwise threshold of $p < 0.001$, and cluster-wise inference using FWE cluster correction $p < 0.05$
Correction	FWE $p < 0.05$

Models & analysis

n/a	Involved in the study
☒	☐ Functional and/or effective connectivity
☒	☐ Graph analysis
☐	☒ Multivariate modeling or predictive analysis

Multivariate modeling and predictive analysis	RSA correlations between brain-based RDMs and model RDMs created from (1) CORnet-S (a four-layer recurrent DNN; Kubilius et al., 2019) and (2) a property-norming study (Devereux et al., 2014).
